# Safety and biodistribution of exosomes derived from human induced pluripotent stem cells

**DOI:** 10.3389/fbioe.2022.949724

**Published:** 2022-08-26

**Authors:** Zhewei Gu, Zhiyu Yin, Pengbo Song, Ying Wu, Ying He, Maoshu Zhu, Zhengxin Wu, Sicheng Zhao, Hongri Huang, Huihuang Wang, Cailing Tong, Zhongquan Qi

**Affiliations:** ^1^ Medical College, Guangxi University, Nanning, China; ^2^ Biotechcomer Co., Ltd., Xiamen, China; ^3^ GuangXi TaiMeiRenSheng Biotechnology Co., LTD., Nanning, China

**Keywords:** exosomes, HiPSCs, safety, biodistribution, nasal administration, Parkinson’s disease

## Abstract

As a new cell-free therapy, exosomes have provided new ideas for the treatment of various diseases. Human induced pluripotent stem cells (hiPSCs) cannot be used in clinical trials because of tumorigenicity, but the exosomes derived from hiPSCs may combine the advantages of iPSC pluripotency and the nanoscale size of exosomes while avoiding tumorigenicity. Currently, the safety and biodistribution of hiPSC-exosomes *in vivo* are unclear. Here, we investigated the effects of hiPSC-exosomes on hemolysis, DNA damage, and cytotoxicity through cell experiments. We also explored the safety of vein injection of hiPSC-exosomes in rabbits and rats. Differences in organ distribution after nasal administration were compared in normal and Parkinson’s disease model mice. This study may provide support for clinical therapy and research of intravenous and nasal administration of hiPSC-exosomes.

## Introduction

When induced pluripotent stem cells (iPSCs) were first established (Takahashi and Yamanaka, 2006), they were a promising cell treatment method in regenerative medicine because of their pluripotency and therapeutic potential for various human diseases. iPSC therapy is currently one of the best options to slow or even stop the progression of Parkinson’s disease (Doi et al., 2020; Schweitzer et al., 2020; Song et al., 2020) because iPSCs selectively differentiate into dopaminergic neurons and have a reduced risk of immune rejection (Deuse et al., 2019; Stoddard-Bennett and Reijo Pera, 2019). Organoids and models derived from iPSCs have provided new ideas for disease treatments and drug screening, and a culture protocol capable of efficiently generating small human brain organoids was optimized to establish subcortical projections in the mouse brain (Dong et al., 2021). The property of differentiation into the three germ layers allows engineering functional tissues (Rao et al., 2018). However, their tumorigenicity is a great challenge in clinical research (Blum and Benvenisty, 2009; Itakura et al., 2017; Liu et al., 2021).

Exosomes can be used as a novel cell-free therapy to resolve issues in cell therapy. The nanoscale particle size of exosomes gives them the ability to cross the blood-brain barrier. Exosomes derived from mesenchymal stem cells pass through the blood-brain barrier and migrate to an injured spinal cord area when administered intranasally (Guo et al., 2019). The combined pluripotency of iPSCs and advantages of exosomes may facilitate development of disease therapies. Adamiak et al. (2018) reported that, among 282 miRNAs detected in iPSCs, 199 miRNAs were also present in iPSC-extracellular vesicles (EVs), indicating that these regulatory transcripts were efficiently transferred from iPSCs to EVs. As a subset of EVs (Kalluri and LeBleu, 2020), iPSC-exosomes may inherit features that exert therapeutic effects on some diseases. To date, there has been no successful strategy that can repair a brain or neural injury and exosomes derived from human induced pluripotent stem cells may be a promising approach (Ghosh et al., 2020).

iPSC-exosomes have specific therapeutic effects on cardiovascular, skin, and eye diseases. Exosomes derived from iPSCs deliver cytoprotective signals to cardiomyocytes to efficiently rescue ischemic cardiomyocytes under conditions such as myocardial ischemia/reperfusion (Wang et al., 2015). iPSC-exosomes may promote the migration of fibroblasts *in vitro* and *in vivo*, and provide a possible treatment for diabetic ulcers (Kobayashi et al., 2018). A corneal epithelial defect model showed that both iPSC- and MSC-exosomes accelerate healing of corneal epithelial defects and the effect of iPSC-exosomes is much stronger than that of MSC-exosomes (Wang et al., 2020). For a promising treatment method, it is crucial to establish the relevant safety evaluation system to facilitate clinical research advances. To accomplish such evaluation, we examined hemolysis, DNA damage, and cytotoxicity of hiPSC-exosomes at the cellular level, their effects on tissues, organs, immunity, and the blood system at the animal level, and their biodistribution.

## Materials and methods

### Statement of ethics and animal treatment

This study was approved by the Animal Studies Committee of Guangxi University (Nanning, China). Animal experiments were carried out in accordance with the committee guidelines. New Zealand rabbits, Sprague-Dawley (SD) rats, and C57 mice purchased from Changsha Tianqing Biotechnology Co., Ltd. (Changsha, China) were fed ad libitum for 1 week for acclimation. Animals were kept in a clean room under stable temperature (22–26°C), humidity (50%–70%), and illumination (12-h light/dark cycle) with water and food freely available.

### Cell culture

hiPSCs purchased from iCell Bioscience Inc., (Shanghai, China) were maintained in mTeSR1 (STEMCELL, #85850) serum-free maintenance medium on Matrigel (Corning, 354277)-coated culture flasks. The medium was replaced with fresh maintenance medium in accordance with cell growth. The culture supernatant was collected for exosome extraction. Mouse macrophage-like cell line RAW264.7 purchased from Biotechcomer Co., Ltd. (Xiamen, China) was maintained in Dulbecco’s modified Eagle’s medium (Gibco, 11965092) containing 10% fetal bovine serum.

### hiPSC identification

Human Embryonic Stem Cell Marker Panel (Hicks et al., 2020; Roth et al., 2020; Bomba et al., 2021; Osnato et al., 2021) (Abcam, ab238602) was used to analyze pluripotency protein markers by immunofluorescence. A Trilineage Differentiation Kit (Ward et al., 2017) (STEMCELL, #05230) was used to induce differentiation into the three germ layers. Human three Germ Layer 3-Color Immunocytochemistry Kit (Han et al., 2020) (R&D, SC022) was used to identify specific protein markers of the three germ layers. A Giemsa Stain solution (Solarbio, G1015) was used to analyze the karyotype. Fluorescence images were obtained under an LSM900 laser scanning confocal microscope (Zeiss, Germany).

### Purification of hiPSC-exosomes

Culture supernatants were stored at 4°C, filtered through a 0.45-μm membrane filter (Millipore, United States), and then concentrated with a 100-kDa molecular weight cutoff hollowfiber membrane. After centrifuging at 2,000 × *g* for 20 min to remove dead cells, the supernatant was centrifuged at 10,000 × *g* for 30 min to remove cell debris. The supernatant was filtered through a 0.22-μm membrane filter (Millipore) and the final supernatant was subjected to ultracentrifugation (Beckman, United States) at 100,000 × *g* for 90 min. All centrifugation steps were at 4°C. The exosome precipitate was resuspended in cold PBS or physiological saline. Exosomes in PBS or physiological saline were filtered again through a 0.22-µm membrane filter. Purified exosomes were stored at −80°C for long-term use or 4°C for short-term use (Cheng et al., 2019; Wu et al., 2021).

### Characterization of hiPSC-exosomes

The protein concentration of hiPSC-exosomes was determined using a BCA protein quantitation kit (Zoman Biotechnology, ZD301). An HT7700 transmission electron microscope (Hitachi, Japan) was used to determine the morphology and size of hiPSC-exosomes. The particle size distribution of hiPSC-exosomes was measured by nanoparticle tracking analysis (Particle Metrix zataview, Germany). Marker proteins of hiPSC-exosomes were detected by western blotting using anti-CD9 (Yang et al., 2022) (Affinity, DF6565), anti-TSG101 (Qi et al., 2022) (Proteintech, 67381-1-Ig), and negative protein marker anti-Calnexin (Sun et al., 2021a) (Proteintech, 66903-1-Ig) antibodies. hiPSC-exosome-specific protein markers CD63 (BD, 556019) and CD81 (BD, 551108) were detected by a Flow NanoAnalyzer (Nanofcm, China).

### Labeling hiPSC-exosomes with PKH26 dye

A PKH26 stock solution (Merck, MINI26) was prepared at 20 μM and mixed at an equal volume with an exosome solution for a final concentration of 10 μM PKH26. After incubation for 1 h at room temperature. the mixture was ultracentrifuged at 100,000 × *g* for 70 min at 4°C to remove excess dye. The precipitate was resuspended in cold PBS(Franzen et al., 2014; Pužar Dominkuš et al., 2018).

### Hemolysis

Twenty milliliters of blood was collected from a New Zealand rabbit, gently stirred to remove fibrin, and then thoroughly mixed with 100 ml physiological saline. After centrifugation at 500 × *g* for 15 min, the supernatant was discarded. The precipitated red blood cells were washed three times with physiological saline until the supernatant was clear. The red blood cell samples were diluted with physiological saline to prepare a 2% suspension and divided into three groups as follows. Negative control group:100 μl suspension with 100 μl physiological saline; Exosome group: 100 μl suspension with 20 μg hiPSC-exosomes in 100 μl physiological saline (final concentration: 100 μg/ml); Positive group: 100 μl suspension with 100 μl distilled water. All experiments were conducted in a 96-well plate. Samples were incubated at 37°C for 3 h and then absorbance was measured at 540 nm in a microplate reader (Tecan, Switzerland).

### DNA damage assay

Leukocytes were isolated from blood using lymphocyte separation medium (Solarbio, P8900) and incubated in a 6-well plate at 37°C for 24 h. Camptothecin (Aladdin, C111281-20mg; 50 μM) was used as a positive control, PBS as a negative control, and 200 μg hiPSC-exosomes (final concentration: 100 μg/ml) as the experimental group. Treated cells were collected and an OxiSelect™ Comet Assay kit (Cell Biolabs STA-350) was used to perform the alkaline comet assay (Singh et al., 1988). The condition for single cell electrophoresis was 1 V/cm (width of electrophoresis tank), electrophoresis time was 20 min, and 300 mA lateral flow electrophoresis was adjusted. Vista Green DNA Dye was used to detect comet its entirety under a fluorescence microscope (Mshot, China). Comet length was analyzed by open comet software (Gyori et al., 2014).

### Cellular uptake and cytotoxicity assays

RAW264.7 cells were seeded in a 6-well plate to prepare for hiPSC-exosome uptake. After washing with PBS, cells were treated with PKH26-labeled hiPSC-exosomes at 37°C for 3 h in serum-free medium (Somiya et al., 2018; Guan et al., 2022). Then, the cells were washed with cold PBS twice and fixed with 4% paraformaldehyde. After mounting on coverslips with anti-fluorescence quenching mounting medium containing DAPI (Solarbio, S2110), the sample was observed under a laser scanning confocal microscope (Olympus, Japan). RAW264.7 cells were seeded in a 96-well plate (2 × 10^4^ cells/well) and treated with up to 200 μg/ml hiPSC-exosomes at 37°C for 24 h. Cell viability was then measured by Cell Counting Kit-8 (Solarbio, CA1210) in accordance with the manufacturer’s instructions.

### Muscle stimulation

Eight healthy New Zealand rabbits weighing approximately 2 kg were selected (equal number of males and females). Negative control group: 200 μl PBS; Exosome groups: 100 and 200 μg hiPSC-exosomes diluted with 200 μl PBS (final concentrations: 500 and 1,000 μg/ml, respectively). Left and right legs were used as experimental and negative control groups, respectively. Animals were acclimated for 1 week before injection. For injection, the needle was inserted vertically into the quadricep muscle in the front of the thigh halfway between the knee and hip joint.

At 48 h after injection, we observed whether the injection site had changed (Sun et al., 2016). After sacrifice by an air injection, two pieces of tissue parallel and perpendicular to the quadricep muscle were collected to perform pathological analysis.

### Vascular stimulation

Eight healthy New Zealand rabbits weighing approximately 2 kg were selected for the experiment (equal number of males and females). Marginal ear vein administration was performed after 1 week of acclimation. Then, 100 and 200 μg hiPSC-exosomes diluted with 200 μl PBS (final concentrations: 500 and 1,000 μg/ml, respectively) were applied to the experimental group, and the negative control received 200 μl PBS. hiPSC-exosomes were injected *via* the left ear marginal vein, and the right ear was injected with the same amount of PBS as the control. Rabbits were injected at the same time for three consecutive days and the location of each injection point was continuously close to the proximal end of the ear. We observed whether blood vessels and surrounding tissues had changed and recorded rectal temperature. Animals were sacrificed at 48 h after the last injection. Surrounding tissues were collected, fixed with 10% formaldehyde solution, and the degree of vascular irritation was evaluated by visual observation and pathological sections.

### Hematology assay

SD rats weighing 250–350 g were selected (15 males and 15 females). A total of 30 rats were divided into five groups with an equal number of males and females in each group. Group 1 rats were subjected to tail vein injection of 200 μl PBS as the negative control for the tail vein group. Group 2 rats were subjected to tail vein injection of 100 μg hiPSC-exosomes diluted with 200 μl PBS (final concentration: 500 μg/ml). Group 3 rats were subjected to tail vein injection of 200 μg hiPSC-exosomes diluted with 200 μl PBS (final concentration: 1,000 μg/ml). Group 4 rats were subjected to nasal administration of 20 μl PBS in left and right nostrils as the negative control for the nasal administration group. Group 5 rats were subjected to nasal administration of 3 × 10^9^ hiPSC-exosomes diluted with 20 μl PBS in left and right nostrils. At days 1, 6, 13, and 20 after administration, 1 ml blood was collected for routine blood examinations using an automatic blood cell analyzer (Mindray, China) and blood biochemistry using an automatic blood biochemical analyzer (Urit, China). Changes in cellular and humoral immunities were analyzed by an Acoustic Focusing Flow Cytometer (Invitrogen, United States) and microplate reader (Tecan). The antibodies used for flow cytometry were anti-CD3 (Invitrogen, 11-0030-82) (Steines et al., 2021), anti-CD4 (Invitrogen, 17-0040-80), anti-CD8 (Invitrogen, 12-0084-82) (Aiello et al., 2017). ELISA kits (Elabscience, IgA E-EL-R3015, IgM E-EL-R3016, and IgG E-EL-R0518c) (He et al., 2019) were used to detect immunoglobulins.

### Parkinson’s disease model

Twelve-week-old C57 male mice were selected for model establishment. Mice were anesthetized with a 2,2,2-tribromoethanol solution before injection with 6-hydroxydopamine (3.3 μg/μl) using a stereotaxic apparatus. Injections were performed at the following two coordinates: anteroposterior (AP), 0.3 mm; mediolateral (ML), −2.2 mm; dorsoventral (DV), −3 mm; AP, 1.1 mm; ML, −1.7 mm; DV, −2.9 mm. The 6-hydroxydopamine solution (2 μl) was injected at each point and the needle was left in place for 5 min to promote drug absorption and prevent reflux. Then, 20,000 IU penicillin was injected for the first 3 days after the operation to prevent surgical infection. One week after surgery, mice received an i.p. injection of apomorphine (0.5 mg/kg). After 5 min of acclimatization, rotational data were continuously recorded for 30 min and the number of revolutions of more than seven circles per minute was considered to be successful model establishment (Pan et al., 2015; Niu et al., 2018; Chen et al., 2020). Additionally, western blotting (Tyrosine Hydroxylase, Abcam, ab75875) (Henriques et al., 2020), HE staining, immunohistochemistry (Tyrosine Hydroxylase, Abcam, ab137869) (Sun et al., 2021b), and Nissl staining were used to verify the reliability of the model.

### Biodistribution of hiPSC-exosomes

Nasal administration of hiPSC-exosomes labeled with PKH26 (excitation: 551 nm; emission: 567 nm) was performed for mouse organ imaging (PerkinElmer, United States; IVIS Lumina LT Series III). The Parkinson’s mouse model established by 6-hydroxydopamine was used as the experimental group and normal mice were the control group. The dose administered to mice was about one-tenth of the number of particles administered to rats in accordance with body weight. A total of 3 × 10^8^ hiPSC-exosomes in 20 μl was administered nasally to each mouse. Mice were deprived of water and food for 12 h before imaging and sacrificed at specific time points for organ fluorescence intensity imaging (Yi et al., 2020). Fluorescence values of each organ were used for analysis.

### Statistical analysis

Statistical analysis was performed using GraphPad Prism v.8.3 for Windows. Intergroup differences were analyzed by the *t*-test for two groups or one-way ANOVA for more than two groups. Data are presented as the mean ± SEM.

## Results

### Typical features of hiPSCs

**FIGURE 1:**
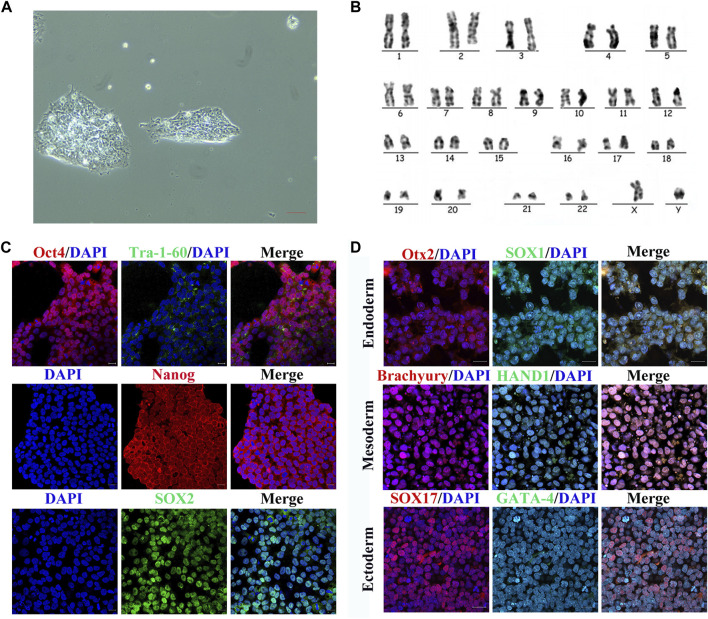
Characterization of hiPSCs. **(A)** Normal monoclonal morphology of hiPSCs in serum-free medium assessed by optical microscopy. Scale bar represents 20 μm. **(B)** Normal chromosomes revealed by karyotype analysis of hiPSCs. **(C)** Immunofluorescence images of pluripotency markers (Oct4, Tra-1-60, Nanog, and SOX2) in hiPSCs. Scale bar represents 50 μm. **(D)** Immunofluorescence images of germ layer markers after hiPSC differentiation. Scale bar represents 50 μm.

To determine whether the cell line had the features of hiPSCs, we assessed the morphological appearance, karyotype, pluripotency markers, and differentiation of the three germ layers. The cells showed colony formation in serum-free medium under trophoblast-free growth conditions (Figure 1A). There were 22 pairs of autosomes and one pair of XY sex chromosomes, which demonstrated a normal karyotype (Figure 1B). Immunofluorescence demonstrated that the cells expressed pluripotency markers Oct4, Tra-1-60, Nanog, and Sox2 (Figure 1C). They had the ability to differentiate into the three germs layers, including endoderm, mesoderm, and ectoderm, as evidenced by expressing Otx2 and Sox1 in endoderm, Brachyury and Hand1 in mesoderm, and Sox17 and Gata-4 in ectoderm (Figure 1D). These findings indicated that the cell line had the characteristics of hiPSCs and could be used in subsequent experiments.

### Characterization of hiPSC-exosomes

**FIGURE 2:**
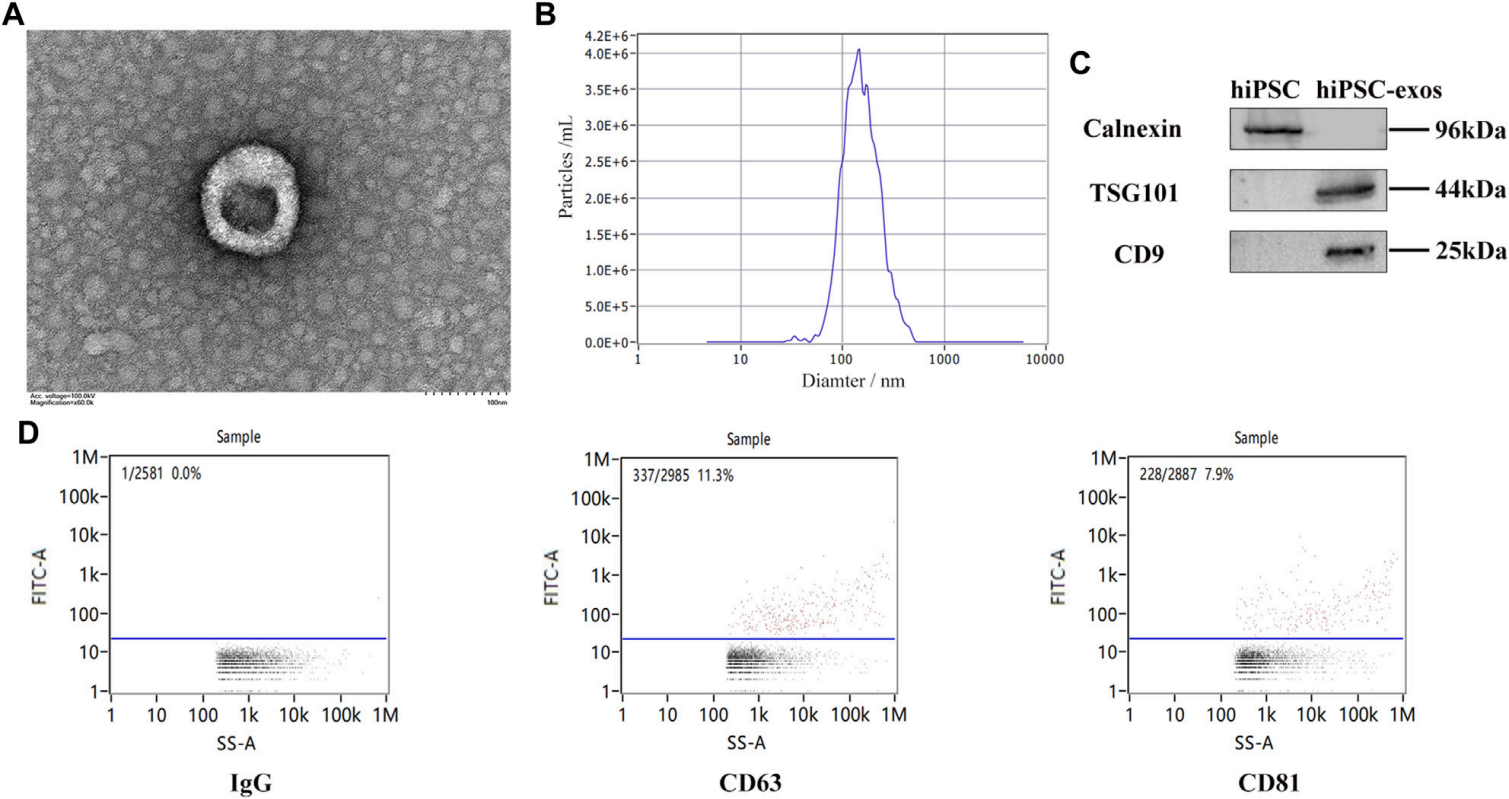
Characterization of exosomes derived from hiPSCs. **(A)** Cup-shaped structure of hiPSC-exosomes under a transmission electron microscope. **(B)** Particle size distribution map of hiPSC-exosomes generated by nanoparticle tracking analysis. **(C)** Specific protein markers (CD9 and TSG101) and negative protein marker (Calnexin) in hiPSC-exosomes detected by western blotting. **(D)** Isotype control IgG and specific markers (CD63, and CD81) in hiPSC-exosomes detected by a Flow NanoAnalyzer.

To evaluate the characteristics, exosomes was isolated from serum-free medium of hiPSCs though ultracentrifugation. Exosomes derived from hiPSCs were cup-shaped under a transmission electron microscope (Figure 2A). The mean diameter of 143.5 nm was observed by nanoparticle tracking analysis (Figure 2B). Typical protein markers CD9 and TSG101 were detected, whereas Calnexin was not detected by western blotting (Figure 2C). Other specific protein markers were detected by a Flow NanoAnalyzer, a novel detection method for hiPSC-exosomes (Figure 2D). These results confirmed the characteristics of hiPSC-exosomes, indicating that the exosomes had been successfully purified and could be used for subsequent cell and animal experiments.

### Hemolytic effect of hiPSC-exosomes

**FIGURE 3:**
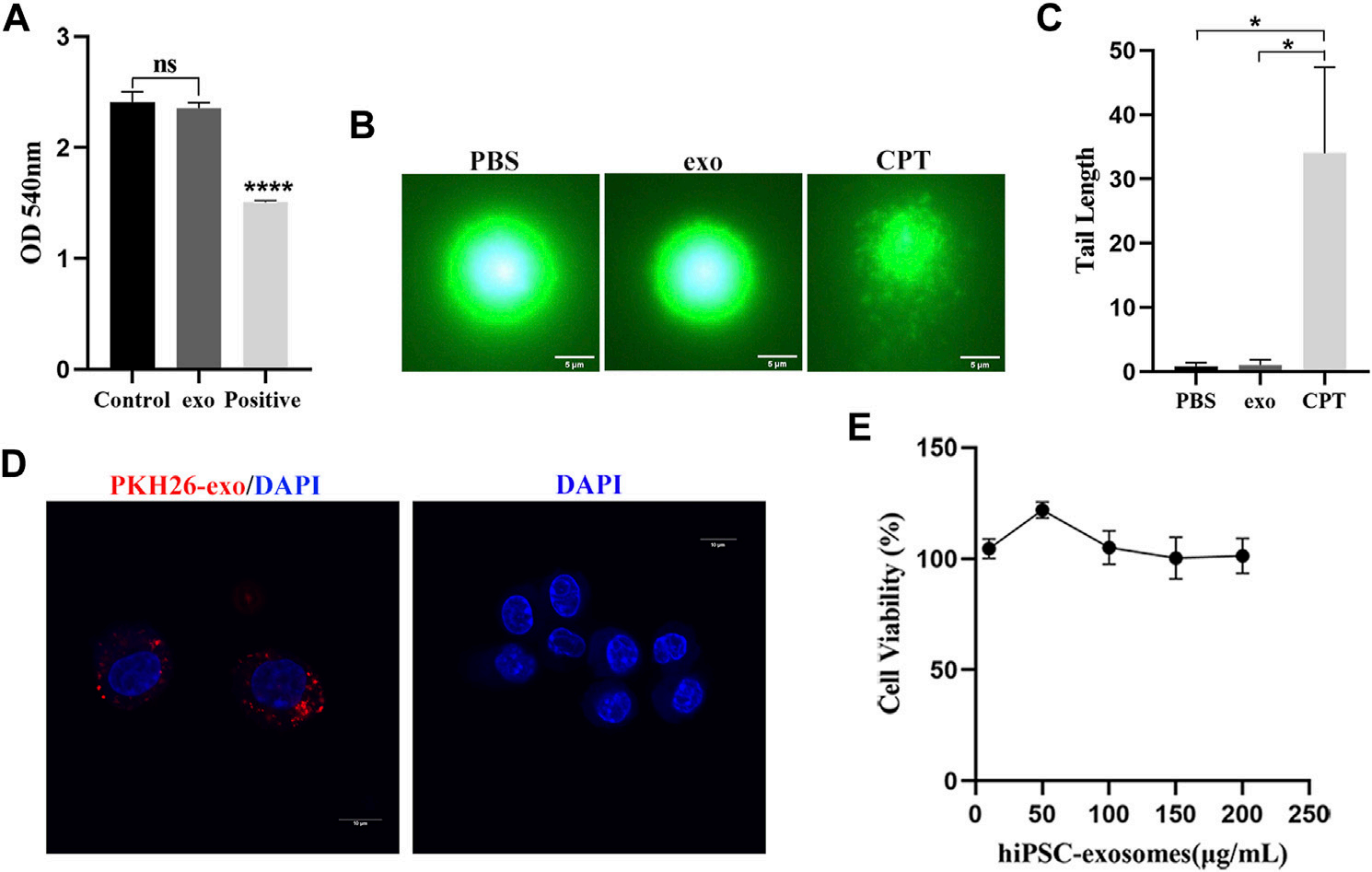
Safety evaluation of hiPSC-exosomes at the cellular level. **(A)** Absorbance values of various groups of erythrocyte suspensions at 540 nm. *N* = 4 per group (ns, no statistical difference; ****p < 0.0001). **(B)** Fluorescence image of DNA after alkaline comet electrophoresis of leukocytes in PBS, hiPSC-exosomes, and the 50 μM camptothecin group. **(C)** Tail length analysis of comets in the three groups. *N* = 5 per group (*p < 0.05). **(D)** PKH26-labeled hiPSC-exosome uptake by RAW264.7 cells under laser scanning confocal microscopy. **(E)** Viability of RAW264.7 cells treated with up to 200 μg/ml hiPSC-exosomes. *N* = 4 per group. Data are expressed as the mean ± SEM.

Because hemoglobin released by hemolysis can affect blood vessels and body systems, we first evaluated the safety of hiPSC-exosomes at the cell membrane. The influence of hiPSC-exosomes on the cell membrane was determined by a red blood cell hemolysis assay. After 3 h of incubation with hiPSC-exosomes, no hemolysis was observed in a red blood cell suspension (Figure 3A). The absorbance of the hiPSC-exosome group was similar to that of the control and showed significant difference from the positive group. Taken together, these finding indicated that hiPSC-exosomes had no effect on hemolysis.

### Assessment of DNA damage caused by hiPSC-exosomes

Next, we performed a comet assay to evaluate the effect of hiPSC-exosomes on DNA. The degree of DNA damage was determined by the length of the comet tail. White blood cells were treated with PBS, hiPSC-exosomes, and CPT and then subjected to single cell electrophoresis. A DNA dye was used to detect the whole comet. Compared with the positive group, PBS and hiPSC-exosome groups did not clearly display the comet tail. Thus, there was no significant effect of hiPSC-exosomes in causing DNA damage (Figures 3B,C).

### Cellular uptake and cytotoxicity of hiPSC-exosomes

We assessed cellular uptake and cytotoxicity to determine the effects of hiPSC-exosomes on cells. Macrophages have ability to phagocytose exosomes (Parada et al., 2021). Mouse macrophage-like cell line RAW264.7 was treated with PKH26-labeled hiPSC-exosomes. Confocal microscopy showed that PKH26-labeled hiPSC-exosomes were phagocytized by RAW264.7 cells (Figure 3D).

RAW264.7 cells were treated with various protein concentrations of hiPSC-exosomes for 24 h. Cell Counting Kit-8 was then used to evaluate cytotoxicity. The cell viability curve showed that the growth status of macrophages was unchanged by hiPSC-exosomes at all concentrations (Figure 3E). The viability of RAW264.7 cells remained at near 100%, even with the highest concentration of 200 μg/ml. These data suggested that the hiPSC-exosomes had no cytotoxicity *in vitro*.

The evaluation of hiPSC-exosomes at the cellular level indicated that the hiPSC-exosomes had no adverse effects on cell membranes, DNA, or cell proliferation.

### Muscle stimulation

To further investigate the safety of hiPSC-exosomes at the animal level, we explored three injection methods to evaluate the influence on various tissues, organs, and systems. We first evaluated the effect of hiPSC-exosomes on muscle tissues by intramuscular injection. Intramuscular injection into quadriceps was performed after the rabbits were acclimated for 1 week. After injection, there was no edema or congestion at the injection sites in hiPSC-exosome and control groups. Samples were collected from the quadricep femoris after sacrificing the rabbits.

**FIGURE 4:**
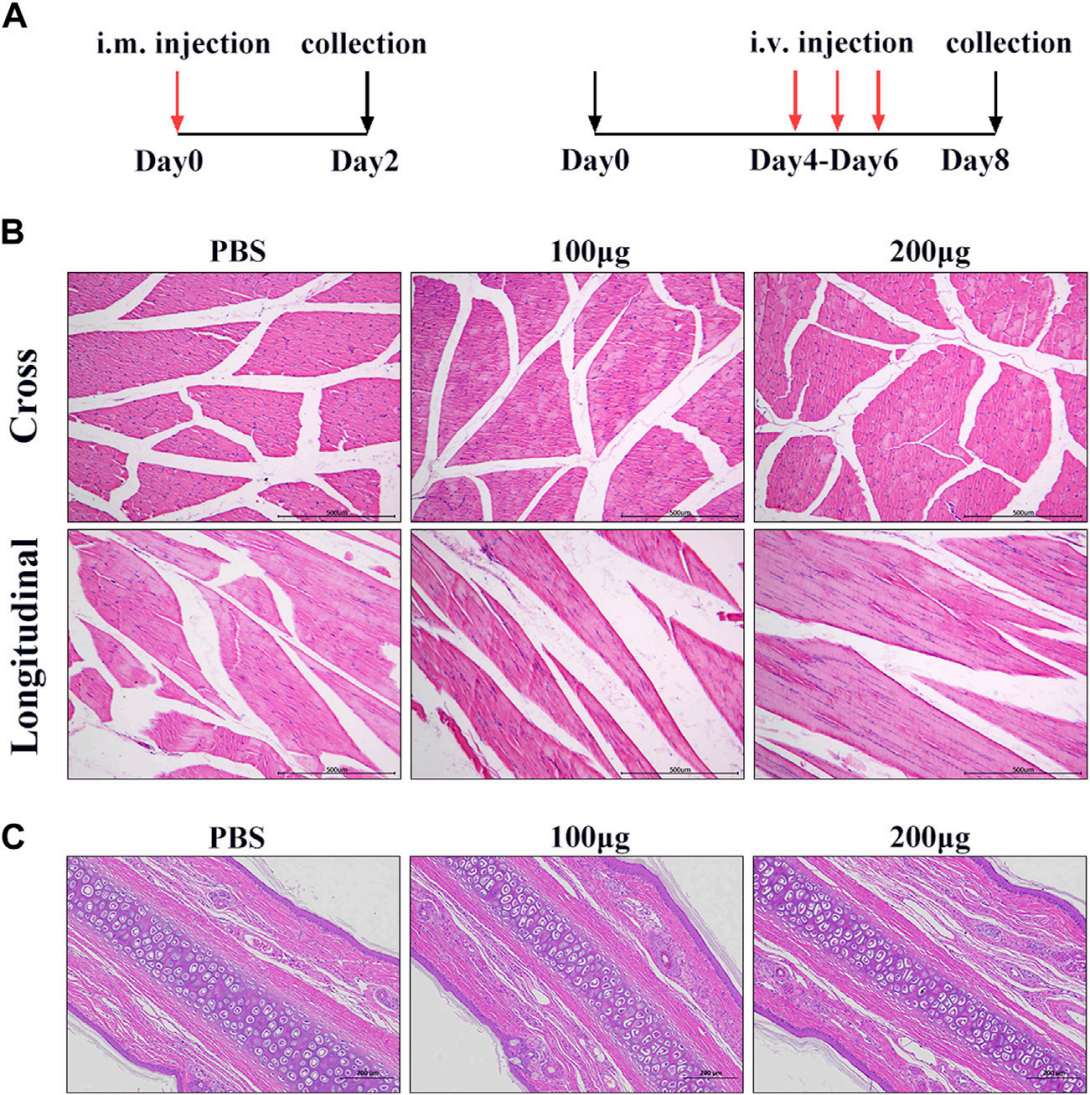
Muscle and vascular stimulation by hiPSC-exosomes. **(A)** Experimental design for injection of hiPSC-exosomes into muscle and vascular stimulation. **(B)** HE staining of muscle in cross-sections and longitudinal sections from control and experimental groups. *N* = 4 per group. **(C)** HE-stained sections of marginal ear veins in control and experimental groups. *N* = 4 per group.

By visual observation and analysis of pathological sections, muscle tissues showed well-defined muscle fiber bundles with a normal form. Muscle cells in the cut surface of muscle tissue were slender and cylindrical multinucleated with different lengths. The nucleus was located near the sarcolemma around the cell, the nucleus was oval, and the nucleolus was obvious. The muscle fibers in the muscle bundles were closely arranged and most of them had an angular appearance without degeneration, necrosis, or inflammatory cell infiltration in hiPSC-exosome groups and showed no significant differences comparing with the control. Taken together, these results showed that i.m. injection of hiPSC-exosomes was safe and hiPSC-exosomes did not stimulate muscle tissues (Figure 4B).

### Vascular stimulation

To determine the effect of hiPSC-exosomes on blood vessels, we performed ear vein injections in New Zealand rabbits. During the administration, ear veins of the rabbits were clear and there was no vascular hemorrhage, congestion, edema, inflammation, tissue necrosis, or other phenomena. At 48 h after the last administration, vascular tissue was collected for sectioning.

H&E staining showed that the morphology of subcutaneous tissue was normal in control and hiPSC-exosome groups. Vascular endothelial cells of the ear vein were arranged normally, skin tissue of the auricle showed slight hyperkeratosis of the epidermis, and no obvious abnormality was observed in the dermis. There was no inflammatory cell infiltration in blood vessels, thickening of the blood vessel wall, or obvious necrosis, degeneration, and inflammation around the wall change (Figure 4C). Similarly, there was no significant difference in the rectal temperature of the rabbits over time in hiPSC-exosome groups compared with the control group (Supplementary Table S1). These results suggested that the injections of hiPSC-exosomes did not affect body temperature and had no stimulatory or adverse effects on vessels. Therefore, we further evaluated other safety through intravenous injection.

### Safety evaluation of hiPSC-exosomes in terms of hemocyte parameters

To confirm whether blood and immunity were influenced by hiPSC-exosomes, we performed hiPSC-exosome administration in rats. Administration of hiPSC-exosomes was divided into three groups including tail vein injection of two protein concentrations and nasal administration (n.a.). The two control groups were administered PBS *via* the tail vein (C1) or nasal cavity (C2). At various days after administrations, blood was collected for analysis.

**FIGURE 5:**
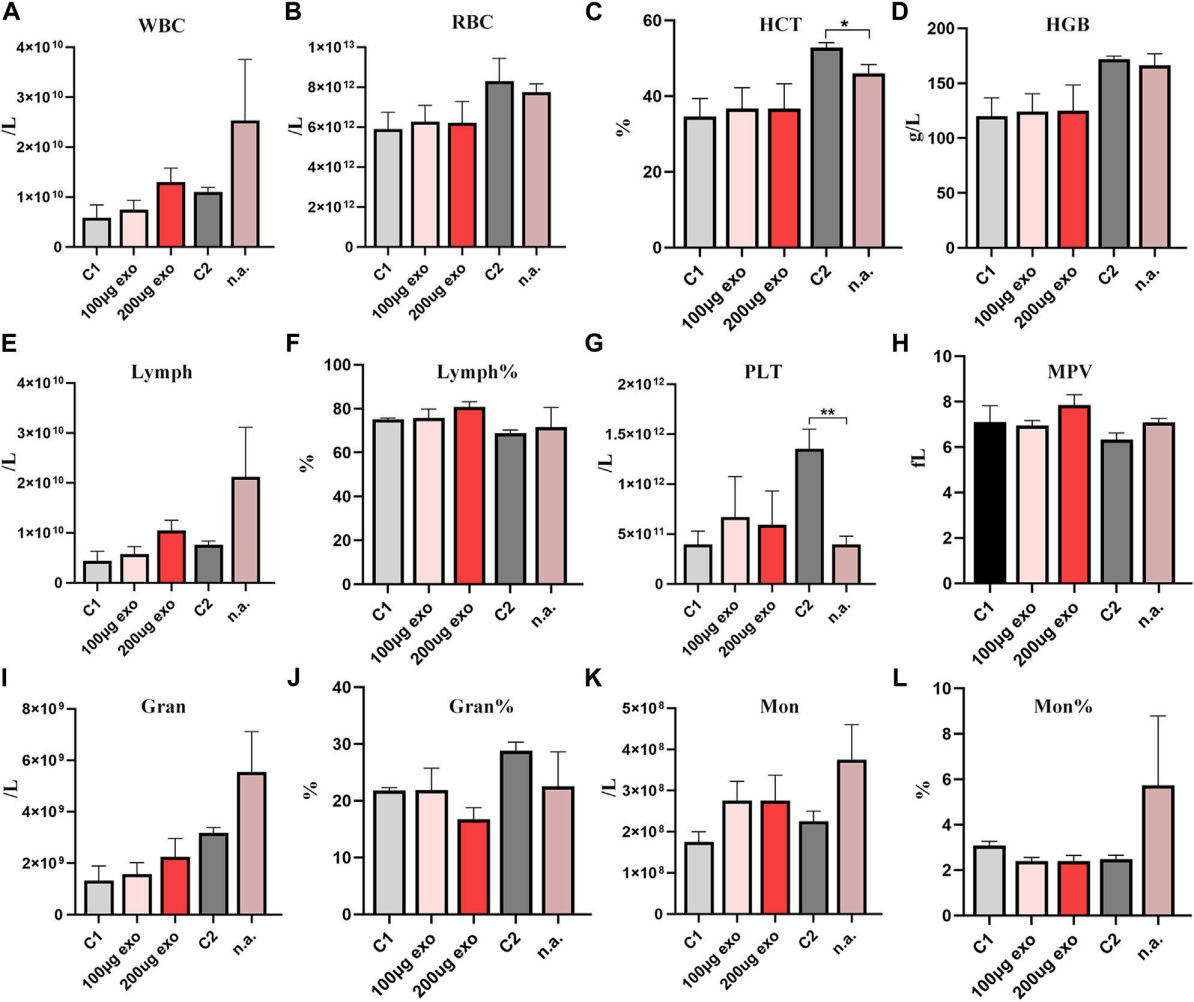
Routine blood analyses of SD rats on day 1. **(A)** White blood cell count. **(B)** Red blood cell count. **(C)** Hematocrit (*p < 0.05). **(D)** Hemoglobin. **(E)** Lymphocyte count. **(F)** % Lymphocytes. **(G)** Platelet count (**p < 0.01). **(H)** Mean platelet volume. **(I)** Neutrophil count. **(J)** % Neutrophils. **(K)** Monocyte count. **(L)** % Monocytes. *N* = 4 per group. Data are expressed the mean ± SEM. (C1, Control 1; C2, Control 2). Reference ranges: WBCs. 2.9–15.3 × 10^9^/L; RBCs, 5.60–7.89 × 10^12^/L; HCT 36%–46%; HGB, 120–150 g/L; Lymph, 2.6–13.5 × 10^9^/L; Lymph%, 63.7%–90.1%; PLT, 100–1,610 × 10^9^/L; MPV, 3.8–6.2 fL; Gran. 0.4–3.2 × 10^9^/L; Gran% 7.3%–30.1%; Mon, 0–0.5 × 10^9^/L; Mon%, 1.5%–4.5%.

The blood was collected from rats for assessment by a hematology analyzer. On day 1 (Figure 5), there was a statistically significant difference in HCT and PLT between C2 and nasal administration groups. On day 6, there was a statistically significant difference in Gran between C2 and nasal administration groups as well as HGB on day 20 (Supplementary Figure S1). RBC showed no significant differences between the groups at the four time points. The number of WBCs and Lymph showed some differences among the groups, but there was no significant difference. There were no significant differences in Lymph% or MPV at each time point. Gran%, Mon, and Mon% showed no significant different at each time. The trend at other times was similar with no significant difference (Supplementary Figure S1). Changes in routine blood indexes were all within the reference ranges of healthy rat indexes.

Thus, we considered that different injection routes may influence some routine blood indexes, but all of them were within the reference range, indicating that tail vein injection and nasal administration of hiPSC-exosomes have no effect on blood cells.

### Safety evaluation of hiPSC-exosomes in the rat liver and kidneys

**FIGURE 6:**
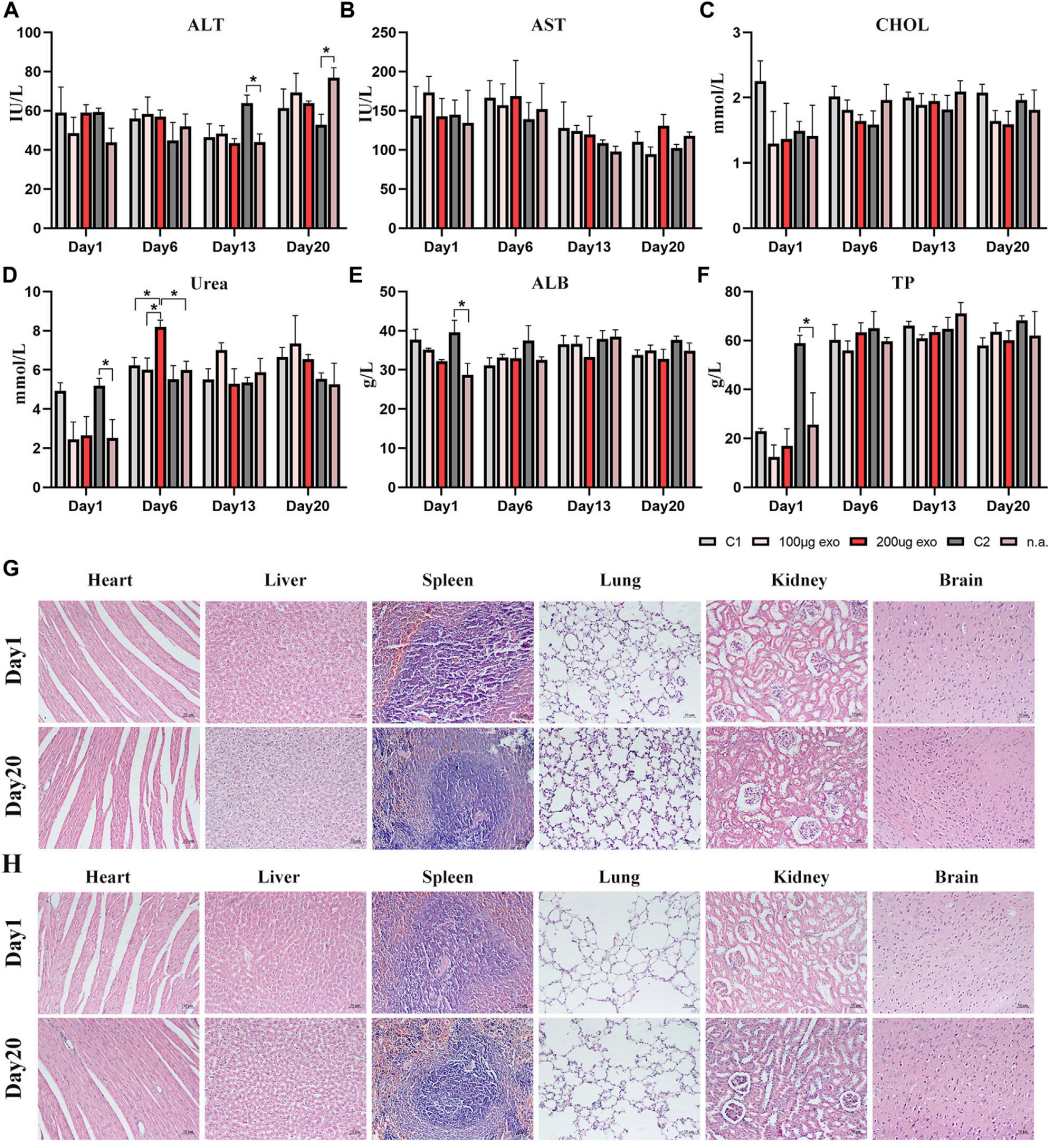
Blood biochemical indexes and pathological changes in rat organs. **(A)** Alanine aminotransferase (*p < 0.05). **(B)** Aspartate aminotransferase. **(C)** Total cholesterol. **(D)** Urea (*p < 0.05). **(E)** Albumin (*p < 0.05). **(F)** Total protein (*p < 0.05). *N* = 4 per group. Data are expressed the mean ± SEM. (C1, Control 1; C2, Control 2). Reference ranges: ALT, 38.84–85.56 IU/L; AST, 75.79–237.34 IU/L; CHOL, 1.05–2.61 mmol/L; Urea, 2.15–8.31 mmol/L; ALB, 30.89–45.08 g/L; TP, 52.41–85.53 g/L. **(G)** HE-stained pathological sections of major organs in the Control 1 group on days 1 and 20. **(H)** HE-stained pathological sections of major organs in the 200 μg hiPSC-exosome group on days 1 and 20.

Next, rat serum was collected to evaluate liver and kidney functions by blood biochemical indexes. Liver and kidney function indicators showed changes in all groups at each time point (Figures 6A–F). ALT content showed significant difference on days 13 and 20 between C2 and n.a. groups. The urea content showed a significant difference on day 1 between C2 and n.a. groups and on day 6 between 200 μg and C1, 100 μg, n.a. groups. ALB and TP contents showed significant differences between C2 and n.a. groups on day 1. There were no significant differences in AST and CHOL. All values evaluated were within the reference ranges. These results suggested that hiPSC-exosomes had no negative effect on the liver and kidney functions of rats.

### Pathological observation of hiPSC-exosomes in various rat organs

Next, we prepared HE-stained pathological sections from rats on days 1 and 20 to investigate whether rat organs had been influenced by the injections (Figures 6G,H). Visual observation and pathological analysis demonstrated no pathological abnormalities or inflammatory cells infiltrates in sections of the heart, liver, spleen, lungs, kidneys, or brain.

### Safety evaluation of hiPSC-exosomes in terms of humoral and cellular immunities in rats

**FIGURE 7:**
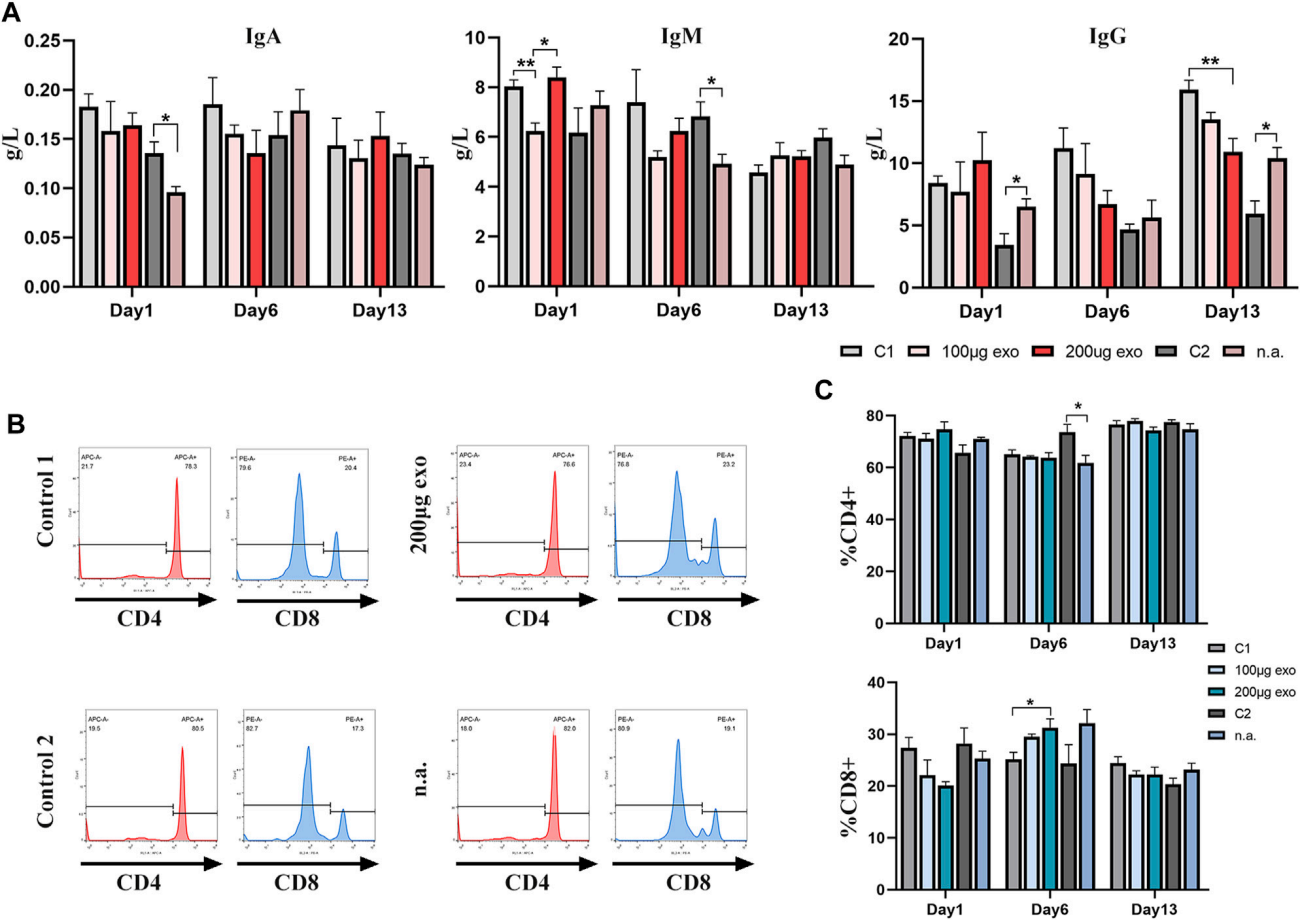
Effects of hiPSC-exosomes on humoral and cellular immunities. **(A)** Changes of IgA, IgM, and IgG contents in the four groups of rats on days 1, 6, and 13. *N* = 4 per group (*p < 0.05; **p < 0.01). **(B)** Representative histograms of tail vein and nasal administration of hiPSC-exosome groups and their control groups in flow cytometry. **(C)** Analysis of changes in the ratios of CD4^+^ and CD8^+^ T-cells on days 1, 6, and 13. *N* = 4 per group (*p < 0.05). Data are expressed as the mean ± SEM.

Effects on humoral and cellular immunities in rats were investigated by ELISAs and flow cytometry. On day 1, IgA contents were different between C2 and nasal administration groups. IgM showed significant differences on day 1 between 100 μg, 200 μg, and C1 groups as well as C2 and n.a. groups on day 6. IgG content showed more obvious differences on days 1 and 13 (Figure 7A).

Flow cytometry was used to measure the ratios of T-cell subsets (Figures 7B,C). The ratio of CD4^+^ T-cells showed a difference on day 6 between C1 and n.a. groups. The ratio of CD8^+^ T-cells between C1 and 200 μg groups showed a difference on day 6.

These data suggested that the influence of hiPSC-exosomes on humoral and cellular immunities reflected by immunoglobulins and T-cell subsets was slight and no obvious trend of negative effects was observed.

Taken together, these findings provided evidence that both administration routes of hiPSC-exosomes were safe in terms of blood components, liver and kidney functions, and organs, and had no adverse trend in humoral and cellular immunities of rats.

### Biodistribution of hiPSC-exosomes in a mouse model of Parkinson’s disease

Next, we evaluated the biodistribution of hiPSC-exosomes in a Parkinson’s disease model in mice. Parkinson’s disease model mice dosed after apomorphine verified for successful model establishment.

**FIGURE 8:**
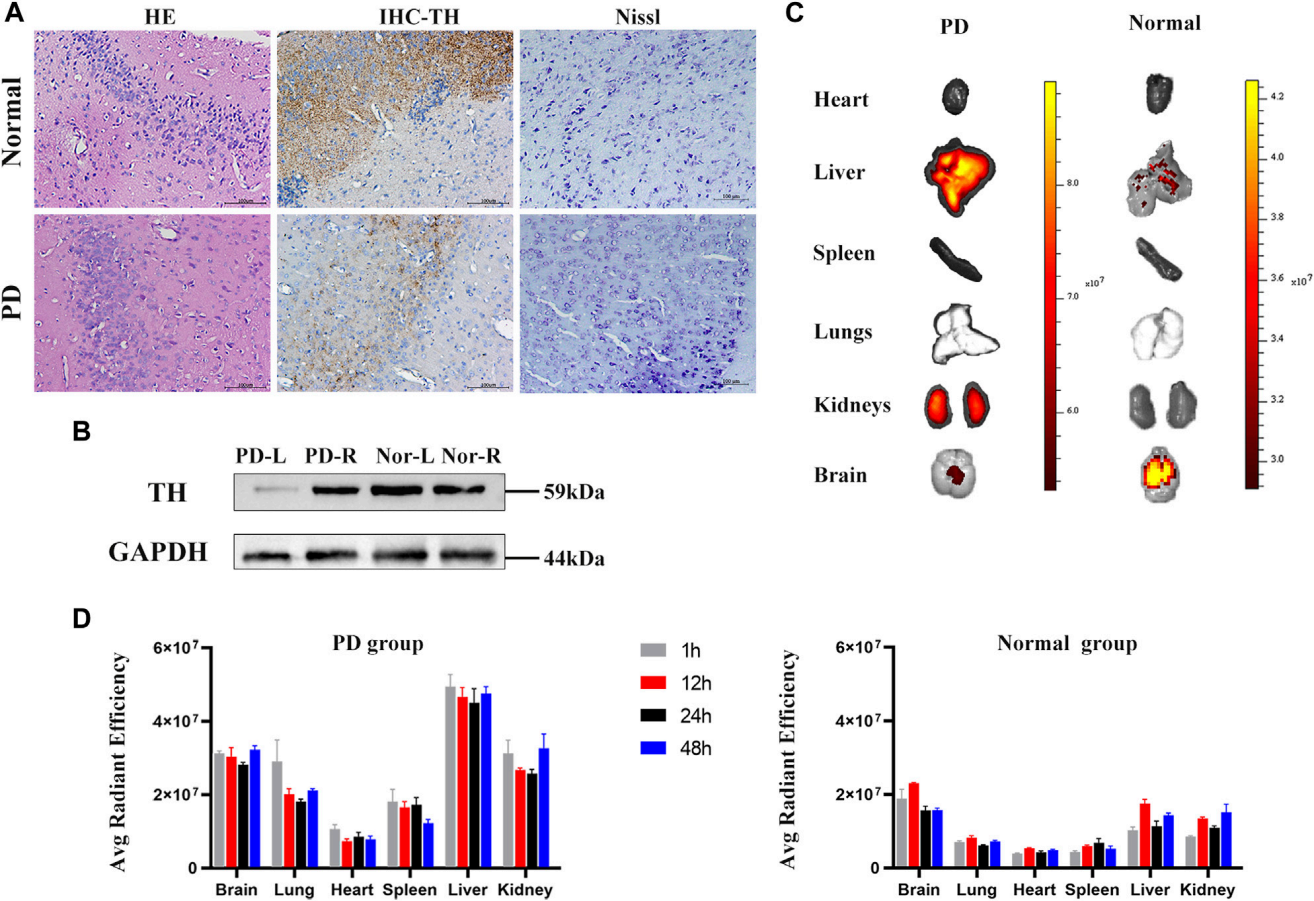
Parkinson’s mouse model test and *in vivo* organ imaging. **(A)** HE staining, IHC of tyrosine hydroxylase, and Nissl-stained sections of control and PD groups. **(B)** Contents of tyrosine hydroxylase in left and right brain hemispheres of PD and control mice measured by western blotting. **(C)** Representative fluorescence images of organs in control and PD groups. **(D)** Fluorescent intensity of major organs at 1, 12, 24, and 48 h. *N* = 3 per group. Data are expressed as the mean ± SEM.

HE pathological sections, immunohistochemistry, and Nissl staining also demonstrated the reliability of the model (Figure 8A). There were differences in the contents of tyrosine hydroxylase in left and right brain hemispheres of control and Parkinson’s disease model mice (Figure 8B). Immediately after sacrificing the mice, organs were harvested for fluorescence imaging.

Fluorescence images showed that labeled hiPSC-exosomes were mainly in the liver, kidneys, brain, and lungs (Figures 8C,D). The tendency of the exosome distribution was essentially consistent with a previous study (Wiklander et al., 2015). The differences between control and Parkinson’s disease model mice were mostly in the brain and liver. Control mice had the highest average fluorescence in the brain, whereas the Parkinson’s disease model mice had the highest average fluorescence in the liver. The average florescence of the PD group was mainly higher than that of the control group. The exosome residence time in both groups was >48 h. Therefore, the labeled hiPSC-exosomes had a different organ biodistribution between control and Parkinson’s disease model mice.

## Discussion

As a new regenerative medicine and drug delivery system, exosomes have received much attention in recent years. hiPSC-exosomes from pluripotent stem cells may inherit their advantages, which may enable more treatment approaches for diseases (Jung et al., 2017; Wang, 2021; Zhou et al., 2021). Exosomes derived from hiPSCs have the potential to treat skin and eye diseases, and especially cardiovascular diseases (Germena and Hinkel, 2021). With the wide applications of hiPSC-exosomes, the establishment of a safety evaluation system is urgent and important to boost the progress of research and clinical trials.

To this end, we evaluated hiPSC-exosomes at the cell level by their influence on the cell membrane, DNA, and cell proliferation as well as the animal leave by their influence on muscle, vessels, the blood system, and humoral and cellular immunities.

First, we identified the characteristics of hiPSCs and their exosomes to ensure the accuracy of experiments. Because exosomes have the same topology as cells (Kalluri and LeBleu, 2020), exosomes pass through the cell membrane by exocytosis and not through channels. Accordingly, the interaction occurs first at the cell membrane. We used red blood cell suspension from rabbits to determine whether exosomes rupture the cell membrane and cause hemolysis. Our results indicated that hiPSC-exosomes did not affect the cell membrane. These results are consistent with a previous study on the hemolytic effect of exosomes derived from mesenchymal stem cells (Sun et al., 2016), which indicated they have the same safety profile in terms of hemolytic effects.

Exosomes enriched with miRNA, RNA, and DNA enter cells and interact with intracellular RNA and DNA (Pegtel and Gould, 2019). To investigate the influence of hiPSC-exosomes on DNA in cells, we performed an alkaline comet experiment. The findings showed that hiPSC-exosomes did not cause DNA damage in cells. A previous study found that EVs from various sources have different effects of DNA (Maji et al., 2017). Our results demonstrated the safety of hiPSC-exosomes in terms of DNA integrity.

Additionally, we assessed their influence on cell proliferation, one of the most important functions of a cell. We first confirmed that the mouse macrophage-like cell line had ability to phagocytose exosomes and the results were consistent with a related study (Xie et al., 2018). Then, we analyzed cell proliferation using Cell Counting Kit-8. The results showed that hiPSC-exosomes slightly promoted cell proliferation. This result is similar to previous findings of EVs (Somiya et al., 2018) which proved their safety in terms of normal cell proliferation. Taken together, we established relevant safety evaluations at the cell level and confirmed that hiPSC-exosomes had no adverse cellular effects.

Exosomes also have close contact with tissues, organs, and systems. For proper evaluation, appropriate methods should be chosen to deliver exosomes *in vivo*. Unlike i.p. injection, i.m. and i.v. injections better describe the effect around the injection site. We chose the quadriceps and ear veins to identify the feasibility of injection methods and effects on the surrounding tissue. The results indicated that both injection approaches were safe and did not cause lesions or inflammatory cell infiltration. Therefore, intramuscular and intravenous injections of hiPSC-exosomes are appropriate for research.

Because the blood system, organs, and humoral and cellular immunities are potential targets of exosomes, we carried out routine blood examinations, blood biochemistry, ELISAs, and flow cytometry for evaluation. Our results demonstrated that i.v. injection and nasal administration did not have adverse effect on blood components, liver and kidney functions, or organs. Some routine blood examinations and blood biochemical indicators showed significant differences at some time points, but all were within the reference ranges of a healthy rat. There were some differences in the relevant indexes of humoral and cellular immunities for both administration routes. We considered that, as a product of human cells, hiPSC-exosomes may be taken up by rat immune cells (Wan et al., 2020) and induce a response by immune cells, which influenced the immune status.

Nasal administration is a novel route of administration, especially for nanoscale particles that may avoid first-pass metabolism and gastrointestinal degradation (Cunha et al., 2017). Our results showed that nasal administration was feasible in most situations with little differences that may cause an immune cell response. A previous study reported that nasal administration might cause an immunoglobulin response (Prado et al., 2008; Jiang et al., 2022), and we also found that nasal administration of hiPSC-exosomes caused a slight immunoglobulin response.

Furthermore, we investigated the distribution characteristics of nasal administration *in vivo*. hiPSC-exosomes had a different biodistribution between control and Parkinson’s disease model mice. The average fluorescence of Parkinson’s disease model mice was overall higher than that in the control group. The largest difference in distribution was found in the brain and liver. A previous study showed that intranasal administration of exosomes leads to a more widespread biodistribution, and in particular, demonstrated enhanced brain accumulation over a long period (Betzer et al., 2017). Our results are consistent with previous studies with hiPSC-exosomes in the control group showing stronger accumulation in the brain. The difference in organs with the highest fluorescence intensity may be caused by modeling. Our data showed that the residence time of hiPSC-exosomes in the body was >48 h, which may facilitate determining when to perform nasal administration.

In conclusion, the safety of intravenous administration of hiPSC-exosomes has been proven at the cell level. Nasal administration as a novel approach to efficiently accumulate exosomes in the brain may be more easily accepted in the clinic. The suitable administration route needs to be determined in accordance with the experimental requirements. The distribution, *in vivo* effects, and pharmacokinetics of hiPSC-exosomes still require be further study.

## Data Availability

The original contributions presented in the study are included in the article/Supplementary Material, further inquiries can be directed to the corresponding authors.

## References

[B1] Adamiak M. Cheng G. Bobis-Wozowicz S. Zhao L. Kedracka-Krok S. Samanta A. (2018). Induced pluripotent stem cell (iPSC)-Derived extracellular vesicles are safer and more effective for cardiac repair than iPSCs. Circ. Res. 122 (2), 296–309. 10.1161/circresaha.117.311769 29118058 PMC5775034

[B2] Aiello S. Rocchetta F. Longaretti L. Faravelli S. Todeschini M. Cassis L. (2017). Extracellular vesicles derived from T regulatory cells suppress T cell proliferation and prolong allograft survival. Sci. Rep. 7 (1), 11518. 10.1038/s41598-017-08617-3 28912528 PMC5599553

[B3] Betzer O. Perets N. Angel A. Motiei M. Sadan T. Yadid G. (2017). *In vivo* neuroimaging of exosomes using gold nanoparticles. ACS Nano 11 (11), 10883–10893. 10.1021/acsnano.7b04495 28960957

[B4] Blum B. Benvenisty N. (2009). The tumorigenicity of diploid and aneuploid human pluripotent stem cells. Cell Cycle 8 (23), 3822–3830. 10.4161/cc.8.23.10067 19887907

[B5] Bomba H. N. Sheets K. T. Valdivia A. Khagi S. Ruterbories L. Mariani C. L. (2021). Personalized-induced neural stem cell therapy: Generation, transplant, and safety in a large animal model. Bioeng. Transl. Med. 6 (1), e10171. 10.1002/btm2.10171 33532581 PMC7823134

[B6] Chen H. X. Liang F. C. Gu P. Xu B. L. Xu H. J. Wang W. T. (2020). Exosomes derived from mesenchymal stem cells repair a Parkinson's disease model by inducing autophagy. Cell Death Dis. 11 (4), 288. 10.1038/s41419-020-2473-5 32341347 PMC7184757

[B7] Cheng Y. Zeng Q. Han Q. Xia W. (2019). Effect of pH, temperature and freezing-thawing on quantity changes and cellular uptake of exosomes. Protein Cell 10 (4), 295–299. 10.1007/s13238-018-0529-4 29616487 PMC6418301

[B8] Cunha S. Amaral M. H. Lobo J. M. S. Silva A. C. (2017). Lipid nanoparticles for nasal/intranasal drug delivery. Crit. Rev. Ther. Drug Carr. Syst. 34 (3), 257–282. 10.1615/CritRevTherDrugCarrierSyst.2017018693 28845761

[B9] Deuse T. Hu X. Gravina A. Wang D. Tediashvili G. De C. (2019). Hypoimmunogenic derivatives of induced pluripotent stem cells evade immune rejection in fully immunocompetent allogeneic recipients. Nat. Biotechnol. 37 (3), 252–258. 10.1038/s41587-019-0016-3 30778232 PMC6419516

[B10] Doi D. Magotani H. Kikuchi T. Ikeda M. Hiramatsu S. Yoshida K. (2020). Pre-clinical study of induced pluripotent stem cell-derived dopaminergic progenitor cells for Parkinson's disease. Nat. Commun. 11 (1), 3369. 10.1038/s41467-020-17165-w 32632153 PMC7338530

[B11] Dong X. Xu S. B. Chen X. Tao M. Tang X. Y. Fang K. H. (2021). Human cerebral organoids establish subcortical projections in the mouse brain after transplantation. Mol. Psychiatry 26 (7), 2964–2976. 10.1038/s41380-020-00910-4 33051604 PMC8505255

[B12] Franzen C. A. Simms P. E. Van Huis A. F. Foreman K. E. Kuo P. C. Gupta G. N. (2014). Characterization of uptake and internalization of exosomes by bladder cancer cells. Biomed. Res. Int. 2014, 1–11. 10.1155/2014/619829 PMC3915764 24575409

[B13] Germena G. Hinkel R. (2021). iPSCs and exosomes: Partners in crime fighting cardiovascular diseases. J. Pers. Med. 11 (6), 529. 10.3390/jpm11060529 34207562 PMC8230331

[B14] Ghosh S. Garg S. Ghosh S. (2020). Cell-derived exosome therapy: A novel approach to treat post-traumatic brain injury mediated neural injury. ACS Chem. Neurosci. 11 (14), 2045–2047. 10.1021/acschemneuro.0c00368 32609493

[B15] Guan P. Liu C. Xie D. Mao S. Ji Y. Lin Y. (2022). Exosome-loaded extracellular matrix-mimic hydrogel with anti-inflammatory property Facilitates/promotes growth plate injury repair. Bioact. Mat. 10, 145–158. 10.1016/j.bioactmat.2021.09.010 PMC8637006 34901536

[B16] Guo S. Perets N. Betzer O. Ben-Shaul S. Sheinin A. Michaelevski I. (2019). Intranasal delivery of mesenchymal stem cell derived exosomes loaded with phosphatase and tensin homolog siRNA repairs complete spinal cord injury. ACS Nano 13 (9), 10015–10028. 10.1021/acsnano.9b01892 31454225

[B17] Gyori B. M. Venkatachalam G. Thiagarajan P. S. Hsu D. Clement M. V. (2014). OpenComet: An automated tool for comet assay image analysis. Redox Biol. 2, 457–465. 10.1016/j.redox.2013.12.020 24624335 PMC3949099

[B18] Han M. J. Annunziata I. Weesner J. Campos Y. Salie M. O'Reilly C. (2020). Generation of human induced pluripotent stem cells (hIPSCs) from sialidosis types I and II patients with pathogenic neuraminidase 1 mutations. Stem Cell Res. 46, 101836. 10.1016/j.scr.2020.101836 32485644 PMC7446138

[B19] He D. Zhang J. Wu W. Yi N. He W. Lu P. (2019). A novel immunodeficient rat model supports human lung cancer xenografts. FASEB J. 33 (1), 140–150. 10.1096/fj.201800102RR 29944447

[B20] Henriques F. Bedard A. H. Guilherme A. Kelly M. Chi J. Zhang P. (2020). Single-cell RNA profiling reveals adipocyte to macrophage signaling sufficient to enhance thermogenesis. Cell Rep. 32 (5), 107998. 10.1016/j.celrep.2020.107998 32755590 PMC7433376

[B21] Hicks D. A. Jones A. C. Corbett N. J. Fisher K. Pickering-Brown S. M. Ashe M. P. (2020). Extracellular vesicles isolated from human induced pluripotent stem cell-derived neurons contain a transcriptional network. Neurochem. Res. 45 (7), 1711–1728. 10.1007/s11064-020-03019-w 32361798 PMC7297870

[B22] Itakura G. Kawabata S. Ando M. Nishiyama Y. Sugai K. Ozaki M. (2017). Fail-safe system against potential tumorigenicity after transplantation of iPSC derivatives. Stem Cell Rep. 8 (3), 673–684. 10.1016/j.stemcr.2017.02.003 PMC5355810 28262544

[B23] Jiang L. Driedonks T. A. P. Jong W. S. P. Dhakal S. Bart van den Berg van Saparoea H. Sitaras I. (2022). A bacterial extracellular vesicle-based intranasal vaccine against SARS-CoV-2 protects against disease and elicits neutralizing antibodies to wild-type and Delta variants. J. Extracell. Vesicles 11 (3), e12192. 10.1002/jev2.12192 35289114 PMC8920961

[B24] Jung J. H. Fu X. Yang P. C. (2017). Exosomes generated from iPSC-derivatives: New direction for stem cell therapy in human heart diseases. Circ. Res. 120 (2), 407–417. 10.1161/circresaha.116.309307 28104773 PMC5260934

[B25] Kalluri R. LeBleu V. S. (2020). The biology, function, and biomedical applications of exosomes. Science 367 (6478), eaau6977. 10.1126/science.aau6977 32029601 PMC7717626

[B26] Kobayashi H. Ebisawa K. Kambe M. Kasai T. Suga H. Nakamura K. (2018). < Editors' Choice > Effects of exosomes derived from the induced pluripotent stem cells on skin wound healing. Nagoya J. Med. Sci. 80 (2), 141–153. 10.18999/nagjms.80.2.141 29915432 PMC5995743

[B27] Liu Q. W. Huang Q. M. Wu H. Y. Zuo G. S. Gu H. C. Deng K. Y. (2021). Characteristics and therapeutic potential of human amnion-derived stem cells. Int. J. Mol. Sci. 22 (2), 970. 10.3390/ijms22020970 33478081 PMC7835733

[B28] Maji S. Yan I. K. Parasramka M. Mohankumar S. Matsuda A. Patel T. (2017). *In vitro* toxicology studies of extracellular vesicles. J. Appl. Toxicol. 37 (3), 310–318. 10.1002/jat.3362 27435060

[B29] Niu J. Xie J. Guo K. Zhang X. Xia F. Zhao X. (2018). Efficient treatment of Parkinson's disease using ultrasonography-guided rhFGF20 proteoliposomes. Drug Deliv. (Lond). 25 (1), 1560–1569. 10.1080/10717544.2018.1482972 PMC6060384 30043675

[B30] Osnato A. Brown S. Krueger C. Andrews S. Collier A. J. Nakanoh S. (2021). TGFβ signalling is required to maintain pluripotency of human naïve pluripotent stem cells. Elife 10, e67259. 10.7554/eLife.67259 34463252 PMC8410071

[B31] Pan X. Chen C. Huang J. Wei H. Fan Q. (2015). Neuroprotective effect of combined therapy with hyperbaric oxygen and madopar on 6-hydroxydopamine-induced Parkinson's disease in rats. Neurosci. Lett. 600, 220–225. 10.1016/j.neulet.2015.06.030 26101828

[B32] Parada N. Romero-Trujillo A. Georges N. Alcayaga-Miranda F. (2021). Camouflage strategies for therapeutic exosomes evasion from phagocytosis. J. Adv. Res. 31, 61–74. 10.1016/j.jare.2021.01.001 34194832 PMC8240105

[B33] Pegtel D. M. Gould S. J. (2019). Exosomes. Annu. Rev. Biochem. 88, 487–514. 10.1146/annurev-biochem-013118-111902 31220978

[B34] Prado N. Marazuela E. G. Segura E. Fernández-García H. Villalba M. Théry C. (2008). Exosomes from bronchoalveolar fluid of tolerized mice prevent allergic reaction. J. Immunol. 181 (2), 1519–1525. 10.4049/jimmunol.181.2.1519 18606707

[B35] Pužar Dominkuš P. Stenovec M. Sitar S. Lasič E. Zorec R. Plemenitaš A. (2018). PKH26 labeling of extracellular vesicles: Characterization and cellular internalization of contaminating PKH26 nanoparticles. Biochimica Biophysica Acta - Biomembr. 1860 (6), 1350–1361. 10.1016/j.bbamem.2018.03.013 29551275

[B36] Qi D. Deng W. Chen X. Fan S. Peng J. Tang X. (2022). Adipose-derived circulating exosomes promote protection of the pulmonary endothelial barrier by inhibiting EndMT and oxidative stress through down-regulation of the TGF-β pathway: A potential explanation for the obesity paradox in ards. Oxid. Med. Cell. Longev. 2022, 1–25. 10.1155/2022/5475832 PMC9098334 35571250

[B37] Rao L. Qian Y. Khodabukus A. Ribar T. Bursac N. (2018). Engineering human pluripotent stem cells into a functional skeletal muscle tissue. Nat. Commun. 9 (1), 126. 10.1038/s41467-017-02636-4 29317646 PMC5760720

[B38] Roth J. G. Muench K. L. Asokan A. Mallett V. M. Gai H. Verma Y. (2020). 16p11.2 microdeletion imparts transcriptional alterations in human iPSC-derived models of early neural development. Elife 9, e58178. 10.7554/eLife.58178 33169669 PMC7695459

[B39] Schweitzer J. S. Song B. Herrington T. M. Park T. Y. Lee N. Ko S. (2020). Personalized iPSC-derived dopamine progenitor cells for Parkinson's disease. N. Engl. J. Med. Overseas. Ed. 382 (20), 1926–1932. 10.1056/NEJMoa1915872 PMC7288982 32402162

[B40] Singh N. P. McCoy M. T. Tice R. R. Schneider E. L. (1988). A simple technique for quantitation of low levels of DNA damage in individual cells. Exp. Cell Res. 175 (1), 184–191. 10.1016/0014-4827(88)90265-0 3345800

[B41] Somiya M. Yoshioka Y. Ochiya T. (2018). Biocompatibility of highly purified bovine milk-derived extracellular vesicles. J. Extracell. Vesicles 7 (1), 1440132. 10.1080/20013078.2018.1440132 29511463 PMC5827637

[B42] Song B. Cha Y. Ko S. Jeon J. Lee N. Seo H. (2020). Human autologous iPSC-derived dopaminergic progenitors restore motor function in Parkinson's disease models. J. Clin. Invest. 130 (2), 904–920. 10.1172/jci130767 31714896 PMC6994130

[B43] Steines L. Poth H. Schuster A. Amann K. Banas B. Bergler T. (2021). Disruption of tfh:B cell interactions prevents antibody-mediated rejection in a kidney transplant model in rats: Impact of calcineurin inhibitor dose. Front. Immunol. 12, 657894. 10.3389/fimmu.2021.657894 34135891 PMC8201497

[B44] Stoddard-Bennett T. Reijo Pera R. (2019). Treatment of Parkinson's disease through personalized medicine and induced pluripotent stem cells. Cells 8 (1), 26. 10.3390/cells8010026 PMC6357081 30621042

[B45] Sun L. Xu R. Sun X. Duan Y. Han Y. Zhao Y. (2016). Safety evaluation of exosomes derived from human umbilical cord mesenchymal stromal cell. Cytotherapy 18 (3), 413–422. 10.1016/j.jcyt.2015.11.018 26857231

[B46] Sun N. N. Zhang Y. Huang W. H. Zheng B. J. Jin S. Y. Li X. (2021a). Macrophage exosomes transfer angiotensin II type 1 receptor to lung fibroblasts mediating bleomycin-induced pulmonary fibrosis. Chin. Med. J. Engl. 134 (18), 2175–2185. 10.1097/cm9.0000000000001605 34483252 PMC8478379

[B47] Sun X. Yu X. Zhang L. Zhao W. Wang M. Zhang Y. (2021b). Comparison of the expression and toxicity of AAV2/9 carrying the human A53T α-synuclein gene in presence or absence of WPRE. Heliyon 7 (2), e06302. 10.1016/j.heliyon.2021.e06302 33665452 PMC7903312

[B48] Takahashi K. Yamanaka S. (2006). Induction of pluripotent stem cells from mouse embryonic and adult fibroblast cultures by defined factors. Cell 126 (4), 663–676. 10.1016/j.cell.2006.07.024 16904174

[B49] Wan Z. Zhao L. Lu F. Gao X. Dong Y. Zhao Y. (2020). Mononuclear phagocyte system blockade improves therapeutic exosome delivery to the myocardium. Theranostics 10 (1), 218–230. 10.7150/thno.38198 31903116 PMC6929612

[B50] Wang A. Y. L. (2021). Human induced pluripotent stem cell-derived exosomes as a new therapeutic strategy for various diseases. Int. J. Mol. Sci. 22 (4), 1769. 10.3390/ijms22041769 33578948 PMC7916646

[B51] Wang S. Hou Y. Li X. Song Z. Sun B. Li X. (2020). Comparison of exosomes derived from induced pluripotent stem cells and mesenchymal stem cells as therapeutic nanoparticles for treatment of corneal epithelial defects. Aging (Albany NY) 12 (19), 19546–19562. 10.18632/aging.103904 33049719 PMC7732275

[B52] Wang Y. Zhang L. Li Y. Chen L. Wang X. Guo W. (2015). Exosomes/microvesicles from induced pluripotent stem cells deliver cardioprotective miRNAs and prevent cardiomyocyte apoptosis in the ischemic myocardium. Int. J. Cardiol. 192, 61–69. 10.1016/j.ijcard.2015.05.020 26000464 PMC4469495

[B53] Ward E. Twaroski K. Tolar J. (2017). Feeder-free derivation of naive human pluripotent stem cells. Stem Cells Dev. 26 (15), 1087–1089. 10.1089/scd.2017.0067 28537496 PMC5563919

[B54] Wiklander O. P. Nordin J. Z. O'Loughlin A. Gustafsson Y. Corso G. Mäger I. (2015). Extracellular vesicle *in vivo* biodistribution is determined by cell source, route of administration and targeting. J. Extracell. Vesicles 4, 26316. 10.3402/jev.v4.26316 25899407 PMC4405624

[B55] Wu J. Y. Li Y. J. Hu X. B. Huang S. Xiang D. X. (2021). Preservation of small extracellular vesicles for functional analysis and therapeutic applications: A comparative evaluation of storage conditions. Drug Deliv. (Lond). 28 (1), 162–170. 10.1080/10717544.2020.1869866 PMC7808382 33427518

[B56] Xie Z. Wang X. Liu X. Du H. Sun C. Shao X. (2018). Adipose-derived exosomes exert proatherogenic effects by regulating macrophage foam cell formation and polarization. J. Am. Heart Assoc. 7 (5), e007442. 10.1161/jaha.117.007442 29502100 PMC5866320

[B57] Yang S. Liu Q. Chen S. Zhang F. Li Y. Fan W. (2022). Extracellular vesicles delivering nuclear factor I/C for hard tissue engineering: Treatment of apical periodontitis and dentin regeneration. J. Tissue Eng. 13, 1. 10.1177/20417314221084095 PMC8935403 35321254

[B58] Yi Y. W. Lee J. H. Kim S. Y. Pack C. G. Ha D. H. Park S. R. (2020). Advances in analysis of biodistribution of exosomes by molecular imaging. Int. J. Mol. Sci. 21 (2), 665. 10.3390/ijms21020665 PMC7014306 31963931

[B59] Zhou Y. Gao Y. Zhang W. Chen Y. Jin M. Yang Z. (2021). Exosomes derived from induced pluripotent stem cells suppresses M2-type macrophages during pulmonary fibrosis via miR-302a-3p/TET1 axis. Int. Immunopharmacol. 99, 108075. 10.1016/j.intimp.2021.108075 34435585

