# Supplementary material for: Safety and biodistribution of exosomes derived from human induced pluripotent stem cells
Source: Front Bioeng Biotechnol. 2022 Aug 26;10:949724. doi: 10.3389/fbioe.2022.949724 (PMC9461140; doi:10.3389/fbioe.2022.949724)
Supplement: Supplementary file 1 [file DataSheet1.docx]

**Supplementary material**


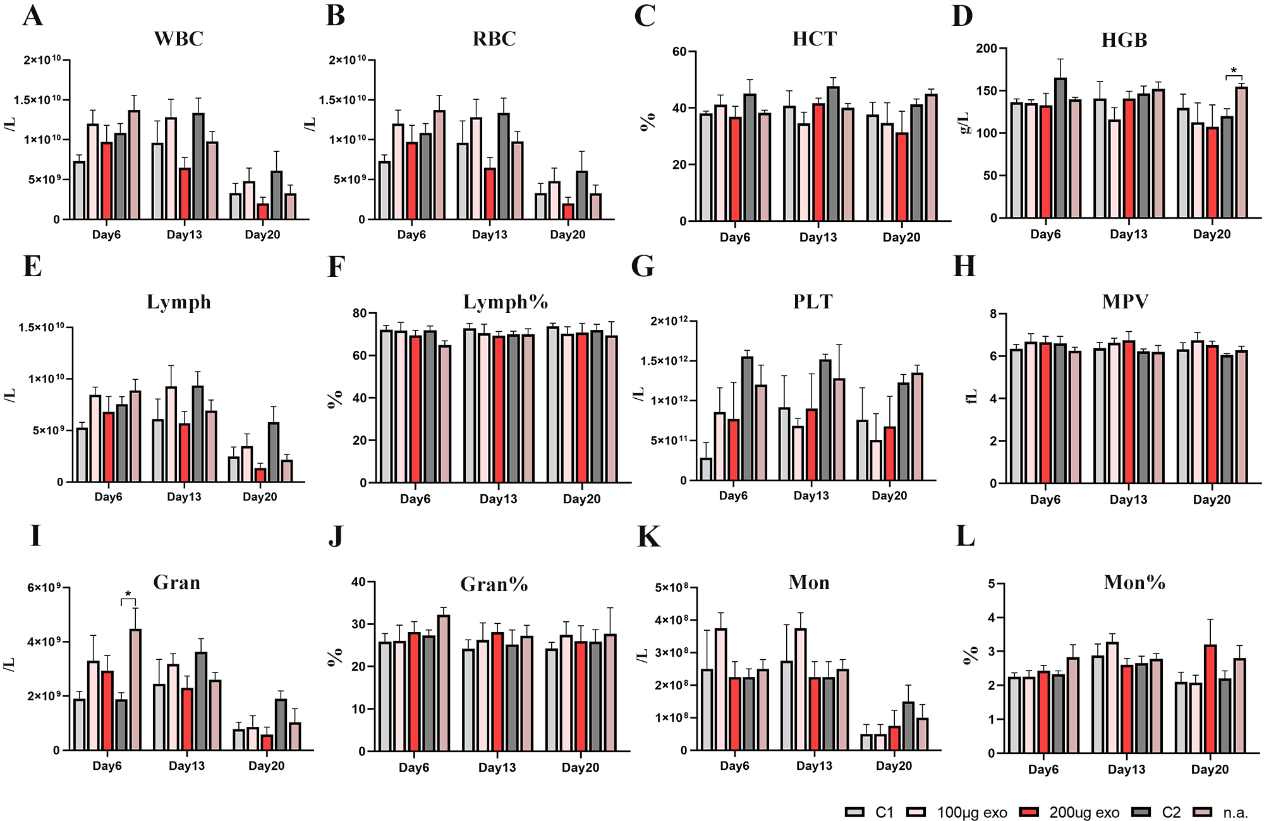


**Supplementary Figure 1. Routine blood analyses in SD rats on days 6, 13 and 20.**

1. White blood cell count. (B) Red blood cell count. (C) Hematocrit. (D) Hemoglobin (*P<0.05).
2. Lymphocyte count. (F) %Lymphocytes. (G) Platelet count. (H) Mean platelet volume.
3. Gran count (*P<0.05). (J) %Gran. (K) Monocyte count. (L) %Monocytes.

N=4 per group. Data are expressed the mean ± SEM. (C1, Control 1; C2, Control 2)

**Supplementary Table 1. Measurement of rectal temperature in experimental rabbits**

| Groups |  |  | Rectal temperature (℃) | | | | | | | | | |  | |
| --- | --- | --- | --- | --- | --- | --- | --- | --- | --- | --- | --- | --- | --- | --- |
|  |  | Day | | 0 | 1 | 2 | 3 | 4 | 5 | 6 | 7 | 8 | |  |
| Normal |  |  | | 40.3 | 40.0 | 40.0 | 40.1 | 39.8 | 40.2 | 39.8 | 40.2 | 39.9 | |  |
| 100 μg exo | |  | | 40.0 | 40.3 | 39.7 | 40.2 | 40.1 | 40.1 | 40.4 | 40.1 | 40.0 | |  |
| 200 μg exo | | | | 40.0 | 39.6 | 39.7 | 39.9 | 39.9 | 39.9 | 40.1 | 40.0 | 39.8 | |  |

N=4 per group. Data expresses the mean rectal temperature of each group.
